# Supplementary material for: Are carbon emissions trading and green financial instruments synergistic? -Comprehensive quantitative research based on content analysis
Source: PLoS One. 2024 Mar 7;19(3):e0298601. doi: 10.1371/journal.pone.0298601 (PMC10919601; doi:10.1371/journal.pone.0298601)
Supplement: S1 Data — (ZIP) [file pone.0298601.s001.zip › Data/地级市Z_F工作报告.zip!/┴╔─■/░░╔╜╩╨/└┤╫╘╣1⁄2╓┌║┼í╛┬φ┐╦╩2╛▌═°í┐_2011.docx]

各位代表：

现在，我代表市政府向大会报告工作，请予审议，并请市政协各位委员提出意见。

一、“十一五”时期主要成就及2010年政府工作回顾

“十一五”时期，在市委的正确领导下，在市人大、市政协监督支持下，市政府认真贯彻落实科学发展观，带领全市人民抢抓机遇，奋勇拼搏，圆满完成了“十一五”规划的各项目标任务。

综合经济实力显著增强。全市地区生产总值增长1.2倍，年均增长17.2%；地方财政一般预算收入增长2.2倍，年均增长26.7%；全社会固定资产投资增长3.8倍，年均增长36.8%，主要经济指标实现了“两个不低于”目标。

城市空间骨架全面拉开。完成新一轮区划调整，打破城市环状封闭结构；建成腾达通海产业大道，构建“一带十区”产业布局；打造“十横八纵五环十射”交通网络，构建区域交通大格局；全面改造“三供”管网，城市综合承载力进一步增强。

产业结构不断优化。鞍钢克服金融危机影响，加快技术改造，推进兼并重组，成功进入世界500强，正在向精品钢基地迈进；地方工业以增量调结构，全面打造钢铁深加工、菱镁新材料、装备制造、化工新材料、光电五大产业集群；加快推进服务业集聚区建设，一批国际知名商业企业进驻鞍山；加快转变农业发展方式，现代农业产业体系初步形成；三次产业结构比重由5.5︰55︰39.5调整到4.3︰53.7︰42，全市经济由主要依靠工业拉动向二、三产业共同拉动转变。

改革开放取得较大突破。不断深化改革，有效解决国有和集体企业历史遗留问题，10.7万名困难企业退休人员纳入基本医疗保险，16.6万名下岗职工重新就业；不断扩大对外开放，全市实际利用外商直接投资增长9.6倍，引进内资增长6.5倍，外贸出口增长2倍。

社会事业全面发展。在全省率先实现每个乡镇都有1所九年一贯制学校，新设7所免费高中，各级各类教育健康发展；在全省率先实施公立医院改革，实行基本药物销售零加价；在全省率先实现每个行政村都有“农家书屋”，城乡群众文化生活不断丰富；在全省率先实现每个社区和行政村都有1处健身场所，鞍山籍运动员在北京奥运会上获得3枚金牌；加大社会事业项目建设投入，开工建设市体育中心、文化中心、职教中心、卫生中心、养老中心。

重点民生工程扎实推进。累计投入100多亿元为城乡群众办实事。全面改造棚户区和危房区，累计建成回迁房685万平方米，回迁居民7.5万户；零就业家庭保持动态为零；强化社会保障体系建设，养老、医疗等社会保险基本实现应保尽保；探索建立困难群体救助保障机制，在全国率先制定出台《城乡特困群众社会救助暂行办法》，城市低保人口由22.1万人下降到6.2万人；实施农村饮水安全工程，农村人口安全饮用水普及率达到87%。城市居民人均可支配收入和农民人均纯收入年均增速均达到14.4%。

“十一五”期间，鞍山先后荣获全国双拥模范城、全国实施畅通工程模范管理城市、全国投资环境百佳城市、全国科技进步先进市、全国人民防空先进市等称号。

各位代表，过去的五年，是鞍山经济实力提升最快、社会建设成效最好的五年；是城乡面貌变化最大、人民群众得到实惠最多的五年。五年发展实践，激发了全市人民加速发展的昂扬斗志，坚定了鞍山老工业基地全面振兴的必胜信心，为鞍山“十二五”时期实现又好又快发展奠定了坚实基础。

各位代表，刚刚过去的2010年，在市委的正确领导下，全市上下深入贯彻落实科学发展观，团结一心，扎实苦干，圆满完成了市十四届人大三次会议确定的各项任务。预计全年实现地区生产总值2200亿元，同比增长20.8%；全社会固定资产投资1500亿元，增长50%；地方财政一般预算收入180亿元，增长25%；城市居民人均可支配收入18514元，增长12%；农民人均纯收入9310元，增长15%；单位生产总值能耗下降5.5%。

工业快速发展。以重点发展主导产业推动工业优化升级，形成五大产业集群；以重大工业项目建设积蓄发展后劲，鞍钢30万吨取向硅钢、鞍钢60万吨精品线材、鞍钢60万吨煤焦油、中船重工100万吨船用钢加工、宝得150万吨热轧H型钢、紫竹80万吨船用钢构、中国软包装集团25万吨双向拉伸聚丙烯薄膜等一大批重大项目开工建设。预计规模以上工业增加值实现990亿元，增长21.6%。

现代服务业快速发展。加快推进站前中央商务区、达道湾现代服务区、达道湾钢材大市场、达道湾汽车贸易产业带、海城西柳商贸城等服务业集聚区建设，红星美凯龙、大商新玛特等85个项目竣工营业。全年新增限额以上服务业企业800家，预计实现社会消费品零售总额515亿元，增长18.9%。招商、浦发、广发、华夏等7家股份制商业银行入驻鞍山，预计全年贷款余额1100亿元，增长16.2%。

县域经济快速发展。现代农业建设步伐加快，全年新发展设施农业30万亩，总面积达到130万亩。大力发展县域主导产业，海城菱镁深加工、台安化工新材料、岫岩非金属矿产品深加工产业集群建设全面推进。加快县城建设，县域地产开发面积达到1106.4万平方米，增长1.92倍。积极应对严重汛情灾害，取得抗洪抢险全面胜利。预计三县（市）地区生产总值、财政一般预算收入增幅均高于全市平均水平。

招商引资成效显著。坚持“走出去，请进来”，全年11次赴日、韩和香港、长三角、珠三角等国家和地区招商引资，成功举办5次大型招商推介会。预计实际利用外商直接投资9亿美元，增长22%；引进内资 700亿元，增长32.1%；外贸出口17亿美元，增长63.6%。

城乡建设全面加强。规划建设达道湾新城、汤岗子新城、海西新城、腾鳌新城和牛庄新市镇、黄沙新市镇、雅河新市镇“四城三镇”。全面建设丹海高速公路，新建和改造腾达通海大道等道路总长166公里，改造虹桥、陈家台桥，开工建设曙光路沙河桥、劳动路地道桥，开通北京航线，开工建设快客铁路鞍山西站、海城西站和牛庄客运站，基本建成鞍山长客北站，建成30个村镇候车站亭。开工建设大伙房水源供水工程，完成引汤入鞍二期供水工程，建成判甲炉污水处理厂。全年植树造林28万亩，新建城市绿地50万平方米，完成矿山植被恢复31.1公顷，城市绿化覆盖率达到39.6%。

社会事业全面进步。鞍山职教城二期工程建成并投入使用，新建和改造69所中小学校。稳步推进公立医院改革和基本药物制度改革，整合职业病医院、传染病医院、结核病医院和精神康复中心，新建骨科医院和肿瘤医院，新建14个社区卫生服务中心。改建电影大厦，新建科技图书馆和10个乡镇综合文化站，市博物馆和文化艺术大厦投入使用。鞍山籍运动员获得广州亚运会7金5银2铜的好成绩。市体育场、体育馆、游泳馆、速滑馆全面建设，乒羽馆投入使用。人口和计划生育、人防和民兵预备役、民族、宗教、外事、侨务、对台、老龄、残联、妇女、儿童、统计、气象、档案、史志等各项工作都取得新成绩。

重点民生工程全面推进。全年实现实名制就业13.7万人，普通高校毕业生就业率达到95.2%，退役士兵技能培训和推荐就业率均实现100%。强化社会保险扩面征缴，确保企业离退休人员养老金按时足额发放，城镇职工及居民医疗保险实现应保尽保，全面启动新型农村社会养老保险试点,新型农村合作医疗实现全覆盖。开工建设动迁周转房50万平方米，建成保障性住房201万平方米，732户农村困难家庭住房问题得到解决。新建养老家园，建成儿童福利院。新增100辆城市公交车，实施铁西部分地区燃气置换工程，免除高中学生扫雪任务，资助6021名困难家庭高中生和大学生，为1.3万名职教城学生提供生活补助。全市重特大安全事故为零，生产安全事故下降25.8%。严厉打击各种犯罪，人民群众安全感进一步增强。

政府行政能力明显提升。自觉接受市人大及其常委会的法律监督、工作监督和市政协的民主监督。认真落实人大议案，全面启动城市垃圾定点定时清运工程，城区环境不断改善。认真落实代表建议和政协提案，实行机关事业单位错时上下班制度，有效缓解交通拥堵状况。坚持市政府常务会议例会制度，推进行政决策科学化、民主化。强化政府制度建设，规范行政权力运行，切实转变工作作风，各级政府争分夺秒，夙兴夜寐，求发展、谋振兴的氛围已经形成。

各位代表，经过全市上下的不懈努力，鞍山已经进入到经济社会又好又快发展的新时期。这些成绩的取得，得益于市委的正确领导和市人大、市政协的大力支持，得益于鞍钢等中省直单位的巨大贡献，得益于各县区、各部门的辛勤工作，得益于全市广大干部的只争朝夕、埋头苦干，得益于全市各族人民的努力拼搏、团结奋斗。在此，我谨代表市政府向为鞍山发展付出艰辛的各位代表和委员，向奋战在全市各条战线的广大干部群众，向各民主党派、工商联、无党派人士、人民团体，向中省直单位、驻鞍部队、武警官兵，向关心支持鞍山发展的老领导、老同志，向所有为鞍山发展作出贡献的海内外朋友，表示衷心的感谢和崇高的敬意！

在肯定成绩的同时，我们也清醒地认识到，我市经济社会发展还存在许多矛盾和问题。一是经济结构性矛盾仍然突出。战略性和牵动性的重大项目少，新兴产业项目不多，特别是节能减排任务艰巨。二是新城规划滞后于新城建设。新城控详规划、分区规划亟待完善。三是涉及群众切身利益的问题还比较突出。冬季供暖、土地征用、房屋拆迁、环境保护、道路交通等方面还存在群众不满意的地方。四是政府自身建设还需进一步加强。一些部门和机关干部思想不解放，加快发展的危机感和责任感不强，办事效率不高、服务意识不强等现象仍然存在。对这些矛盾和问题，我们必须以对鞍山发展高度负责的精神，采取更加有力的措施，认真加以解决，决不辜负全市人民的期望。

二、“十二五”时期主要任务和2011年政府重点工作

各位代表，回首刚刚过去的五年，鞍山站在了一个新的历史起点；展望“十二五”新的征程，我市经济社会发展任重道远。在市委的领导下，市政府组织编制了《鞍山市国民经济和社会发展第十二个五年规划纲要（草案）》，提请本次会议审议。

“十二五”时期我市经济社会发展的总体思路：全面贯彻党的十七大和十七届三中、四中、五中全会精神，深入贯彻落实科学发展观，抓住东北振兴和辽宁沿海经济带、沈阳经济区上升为国家战略的双重机遇，按照“围绕一个核心，加速两个融入，推进三大任务”的总体要求，以科学发展、创新发展、和谐发展为主题，以转方式、调结构为主线，以项目建设为核心，以推进工业化、城市化为重点，以改革开放为动力，切实加强节能减排和生态环境保护，着力保障和改善民生，促进社会和谐稳定，持续推进鞍山经济社会又好又快发展。

“十二五”时期全市经济社会发展的主要目标：经过五年努力，把鞍山打造成为世界级精特钢和钢铁深加工基地、世界级菱镁新材料产业基地、亚洲温泉旅游城、东北钢铁物流中心。全市地区生产总值、固定资产投资和地方财政一般预算收入实现“三个翻番”，城市居民人均可支配收入和农民人均纯收入力争实现翻番。

全面优化产业结构。把工业结构优化升级作为发展现代产业体系和调整产业结构的重点，改造提升传统产业，培育发展战略性新兴产业，大力发展产业集群。到2015年末，形成精特钢和钢铁深加工、菱镁新材料、装备制造3个千亿元以上产业集群，形成化工新材料、光电2个300亿元以上产业集群，全市规模以上工业销售收入达到6000亿元，工业增加值达到2000亿元。把加快发展现代服务业作为推动产业结构优化升级的重要支撑，优先发展生产性服务业，重点发展生活性服务业，大力发展社会性服务业。到2015年末，形成8个销售收入50亿元以上的中心商务集聚区、4个销售收入100亿元以上的物流集聚区、2个旅游收入100亿元以上的文化旅游集聚区。把发展民营经济作为产业结构优化升级的重要着力点，支持民营资本拓宽投资领域，重点发展新材料、新能源、高端制造业、高端服务业等产业。到2015年末，形成10户销售收入100亿元以上企业，100户10亿元以上企业，1000户亿元以上企业和1万户千万元以上企业。

全面构建县域发展格局。把加快发展设施农业和农产品加工业、流通业作为发展现代农业的重点，培育各具特色的农业主导产业集群。到2015年末，将海城打造成为“四辣”产业基地，将台安打造成为鸭鹅产业基地，将岫岩打造成为蘑菇产业基地。以县域工业园区为载体，构建县域工业主导产业集群。到2015年末，海城形成超千亿元的菱镁新材料产业集群，台安形成超百亿元的化工新材料产业集群，岫岩形成超百亿元的非金属矿产品深加工产业集群。以县城改造和新城新市镇建设为重点，加快推进农村城市化、城镇化。到2015年末，海城市建成70万城市人口的中等城市，力争步入全国百强县前十名；台安县建成15万城市人口的中小城市；岫岩县建成20万城市人口的生态宜居城市；达道湾、汤岗子、腾鳌、海西新城和牛庄、黄沙、雅河新市镇基本建成。

全面构筑城市发展格局。把“提升主城，建设新城，突出特色，全面发展”作为科学规划城市空间布局的发展战略，加快形成西部工业产业带、中部都市生活带、东部生态旅游带的发展格局。铁东区、铁西区重点发展现代服务业，立山区、达道湾新城重点发展工业和现代服务业，千山区重点发展温泉休闲康复旅游业，高新区重点发展战略性新兴产业，千山风景区、鞍山文化旅游开发区和玉佛山风景区重点发展文化旅游业。完善城市基础设施，提高城市承载力。全面完善公路、铁路、城际轨道、航空等综合交通体系，建设东北重要交通枢纽城市；推动热电、水利、供电、供水、燃气等重大项目建设，提高城市综合服务功能。

全面改善城乡环境。把构建资源节约、环境友好的生产方式和消费模式作为可持续发展的重点，加大节能减排力度，强化环境综合整治，加强生态保护和恢复，不欠新账、多还旧账，造福社会、惠及子孙。到2015年末，单位生产总值能耗及水耗、化学需氧量、二氧化硫排放量等指标达到国家及省要求，主要污染物排放总量下降10%左右，城市污水处理率达到90%以上，生活垃圾无害化处理率达到100%，城区全部实现集中供热，城市绿化覆盖率达到42%以上，全地区森林覆盖率达到50%，让鞍山的天更蓝、水更清、山更绿、环境更美。

全面完善公共服务体系。把发展社会事业、着力保障和改善民生、推进基本公共服务均衡化、为鞍山人民创造更多福祉作为政府工作的重要任务。到2015年末，健全覆盖城乡的公共教育服务体系、公共医疗卫生服务体系、公共文化服务体系、全民健身服务体系、社会管理服务体系和社会治安防控体系，健全覆盖城乡居民的养老保险体系、基本医疗保险体系和弱势群体基本生活保障体系，让发展振兴的成果更多地惠及百姓，让鞍山人民生活得更加幸福、更加美好。

各位代表，2011年是“十二五”开局之年。我们将按照市委的总体要求，围绕“十二五”规划纲要确定的奋斗目标和主要任务，坚持一手抓发展、一手抓民生，一手抓城市、一手抓农村，一手抓空间、一手抓项目，解放思想，开拓创新，扎实苦干，保持经济社会又好又快发展态势，为“十二五”打下坚实基础。主要预期目标：地区生产总值增长15%；全社会固定资产投资增长20%；地方财政一般预算收入增长16%；城市居民人均可支配收入增长13%，农民人均纯收入增长14%。

按照上述目标，我们将重点抓好以下八个方面工作：

（一）以增量调结构，牢固树立工业大市形象

全面实施“工业五项工程”。按照省委、省政府的要求，全力推进企业升级、项目建设、产业集群、企业并购、淘汰落后“工业五项工程”建设。支持骨干企业发展，重点扶持超10亿元企业做大做强。每个县（市）区和经济开发区都要引进一批投资超10亿元、50亿元，乃至100亿元的重大工业项目，为鞍山未来发展提供强力支撑。大力发展战略性新兴产业，推进五大主导产业集群建设，全年并购海内外企业6户。全力跟踪重点项目，推进项目落地和开工建设，年内开工千万元以上工业项目600个，实现规模以上工业增加值增长15%以上。

全力支持鞍钢等中省直企业发展。鞍山因钢而立，因钢而兴。要叫响“钢都”品牌，依托鞍钢打造世界级钢铁深加工基地，支持鞍钢打造世界级精品钢基地，帮助鞍钢早日建成特大型跨国集团。支持中国三冶、中冶焦耐、中冶北方、中钢热能等中直企业优化产业结构，做大做强总部经济。

大力扶持民营经济发展。鼓励和支持中小企业通过“上船重组、上市融资、上档改造”，提升企业，壮大企业。强化政策支持，拓宽融资渠道，推进技术创新，优化服务环境，促进民营经济快速发展，全年民营经济增加值增长20%以上。

（二）大力发展现代服务业，培育新的经济增长点

加快发展商贸流通业。优化商贸布局，打造商务集聚区和物流集聚区。重点推进铁东站前中央商务区、铁西人民路商务区、立山曙光路商务区、汤岗子温泉城商务区、高铁客运站商务区、海西新城商务区等中心商务集聚区建设，重点推进达道湾批发大市场、达道湾钢材大市场、立山五金机电大市场、达道湾汽车贸易产业带、海城西柳商贸城、南台皮革皮具城、海城水暖城等大市场建设，全市社会消费品零售总额增长17%以上。

加快发展文化旅游业。依托千山、温泉、岫玉、钢都旅游资源，全力打造千山、汤岗子两大旅游集聚区，重点推进单体投资百亿元以上的港中旅温泉旅游城、奥特莱斯商城、欢乐世界主题公园、千山影视基地、东四方台温泉世界等一大批精品文化旅游项目建设，力争用五年时间将鞍山打造成为旅游目的地和旅游强市。

大力发展金融业。鼓励金融机构扩大信贷投放，力争新增贷款增幅处于全省前列。鼓励金融企业在鞍设立区域总部、分支机构。鼓励企业进入资本市场，年内实现2户企业上市，做好10户企业上市准备。完善金融服务体系，拓宽融资渠道，大力发展投资基金公司、村镇银行、担保公司和小额贷款公司，力争年内在全省率先设立投资基金公司。做大做强市商业银行，力争年内成为区域性银行。

（三）围绕三年翻番目标，加快发展县域经济

推进农业产业化。按照“一县一业”、“一县一品”的要求，重点扶持农业产业化龙头项目，大力推进海城“四辣”、台安鸭鹅、岫岩食用菌农业主导产业集群发展。切实加强蔬菜基地建设，提高地方蔬菜供给能力。大力发展农民专业合作组织，拓宽农民增收渠道，巩固和扩大设施农业比重，全年新增设施农业面积20万亩。加强农业基础设施建设，强化中小河道和水毁工程治理。

推进农村工业化。加大县域工业园区投入，完善园区基础设施，重点扶持重大工业项目建设，加快推进海城菱镁新材料、台安化工新材料、岫岩非金属矿产品深加工产业集群建设，全年县域开工千万元以上工业项目400个。

推进农村城镇化。科学规划县城，高标准建设县城。按照政府主导，市场化运作的原则，积极引进战略投资者，加快新城新市镇建设。坚持统筹规划，依法用地，节约集约用地，积极稳妥推进农村土地整治，完善农村集体经营性建设用地流转，切实保障被征地农民合法权益。

（四）扩大改革开放，增强地区经济发展活力

加大深化改革力度。建立规范的国有资产监督管理体制，完善企业法人治理结构，盘活闲置国有资产，以存量吸引增量。下放管理权限，赋予高新区、达道湾开发区市级管理权限，赋予城区县级管理权限，赋予中心镇县级管理权限，力争达道湾开发区进入国家级开发区，台安九股河开发区、岫岩雅河开发区进入省级经济开发区。加快推进公立医院改革，走出一条可持续、可借鉴、有示范作用的新路子。

加大招商引资力度。坚持招商与选商相结合，重点推进具有牵动作用，支撑未来经济发展的工业大项目、商业大项目、旅游业大项目，特别是战略性新兴产业大项目。突出主题概念、产业集群招商，依托鞍钢资源对外招商，鼓励地方企业重组招商。重点面向香港、长三角、珠三角等资本密集区和行业龙头企业、中央大企业、世界500强企业，开展点对点、面对面招商。全年实际利用外商直接投资增长15%以上,引进内资增长30%以上。

加大向上争取资金和政策工作力度。及时把握国家政策，用好用足国家政策，研究申报一批符合国家产业目录和资金投向的项目，积极争取国家和省资金、政策支持，确保争取国家和省资金额度在全省各市平均水平以上。今后政府新建项目所需资金，原则上以向上争取资金和市场化运作为主，以财政资金或贷款配套为辅，实现负债额度与偿债能力的总体平衡。

（五）科学规划建设，全面改善人居环境

坚持高起点规划。按照“先规划，后建设”的原则，加快城市总体规划修编，编制新城规划、分区规划和专项规划，提高控详规划覆盖率，使城市每个地块都列入规划，每个项目都按照规划实施。搞好城市空间形态、建筑风格与色彩设计，全面提升城市品质。严格规划审批和监管，坚决维护规划的严肃性和权威性。

坚持高标准建设。全面推进汤岗子新城、达道湾新城，千山文化旅游带、万水河景观带“两城两带”建设，高水平规划建设深沟寺和高铁站前周边地区。加快推进千山东路等17条市区道路建设，建设鞍台高速公路，续建丹海高速公路，开工建设沈西工业走廊通海产业大道鞍山段，完成曙光路沙河桥等62项市政道路桥梁工程。启动市动物园改造工程。

坚持高水平管理。按照规范化、系统化、精细化原则，全面加强城市管理。充分发挥城区政府职能作用，推进城市管理重心下移。综合整治市容市貌，重点对马路市场、站前广场、社区巷道、人行步道、广告牌匾、环境卫生、工地扬尘、违章建筑、非法营运、道路交通等10个方面进行专项整治，让鞍山市民生活在舒适的城市环境中。

高度重视生态环境保护和治理。坚持保护优先和自然恢复为主，从源头上扭转生态环境恶化趋势。全力支持鞍钢实施5台烧结机脱硫设施项目建设，新建2座35万千瓦和2座30万千瓦热电联产项目，新建3座污水处理厂，新建1座垃圾处理厂，规划建设再生资源产业园和污泥处理中心。积极争创国家森林城市，实施城市周边矿山生态恢复工程，强化台安西北部沙化治理，对100个村庄实施连片综合整治，全年植树7000万株，造林36万亩，新增城市景观绿化90万平方米。按照国家、省标准控制主要污染物排放总量，力争城区85%以上天数的空气环境质量达到国家二级标准。

（六）统筹兼顾，协调发展各项社会事业

优先发展教育事业。扩大学前教育资源，筹建公立幼儿教育集团，鼓励社会资本兴办幼儿教育，努力解决“入园难、入园贵”问题。改善办学环境，新建10所中小学校，新建和改造4所高中。大力发展职业教育，力争将职教城纳入国家职业教育基地。积极为辽宁科技大学、鞍山师范学院发展和重点学科建设创造良好条件。

加快发展卫生事业。健全公共卫生服务体系，提升基本医疗服务能力，努力解决“看病难、看病贵”问题。深化医药卫生体制改革，推进公立医院改革。整合医疗资源，实现医疗机构集团化、网络化，年内将铁西医院、鞍山二院整合为中国医大第一医院集团附属医院。鼓励社会资本进入医疗服务市场，吸引民营和境外资本发展专科医院。

加快发展文化体育事业。坚持一手抓公益性文化事业、一手抓经营性文化产业。加快推进胜利会堂改造和社区文化中心等文化设施建设，不断丰富人民群众文化生活。鼓励和引导非公有制经济发展新型文化产业，推动文化产业成为我市经济支柱性产业。开工建设市民健身服务中心，建成市体育场、体育馆、游泳馆和速滑馆。

继续做好人口和计划生育、民族、宗教、外事、侨务、老龄、残联、妇女儿童、优抚安置、防灾减灾、国家安全和双拥共建等各项工作。

（七）保障和改善民生，促进社会和谐发展

全力稳定和扩大就业。坚持以创业带动就业，推进大学生、退役士兵、新生劳动力和就业困难群体就业。强化普惠制培训，培养更多适用型人才，缓解就业结构性矛盾。全年实名制就业10.4万人，城镇登记失业率控制在3%以内，保持零就业家庭动态为零。

继续完善社会保障体系。强化社会保险扩面征缴，实现应收尽收、应保尽保，确保企业离退休人员基本养老金按时调增和足额发放。积极推进岫岩县新型农村社会养老保险试点。进一步扩大城镇居民基本医疗保险覆盖面，提高医疗保险待遇水平。强化低保动态管理，提高城乡低保户和五保户保障标准。加快养老家园建设，大力推进社会养老事业和慈善事业发展。

切实改善群众生活条件。继续为城乡群众办实事。大力推进保障性住房建设和农村危房改造，决不让困难家庭因贫而无房居住。建立和完善城乡困难家庭子女助学体系，决不让困难家庭子女因贫而失学。实施“蓓蕾慈善救助工程”，决不让困难家庭先天性心脏病、唇腭裂儿童因贫而终身残疾。实施“夕阳慈善救助工程”，决不让困难家庭白内障老人因贫而失去光明。强化价格调控监管，适时增加困难群体生活补贴，决不让困难家庭因物价上涨而降低生活水平。严厉打击生产销售假冒伪劣食品和药品等违法行为，决不让假冒伪劣食品、药品损害人民群众身体健康。

大力推进和谐鞍山建设。以政府信用、企业信用和个人信用体系建设为重点，加快“信用鞍山”建设，形成诚实守信的社会道德风尚。加强社区管理，创建和谐社区。加强社会治安综合治理，全面完善社会治安防控体系，不断增强人民群众安全感。加强群众来信来访工作，妥善处理社会矛盾，切实解决人民群众关心的热点难点问题。加强安全生产和消防安全，坚决遏制重特大安全事故发生。

（八）切实加强政府自身建设，全力打造为民务实高效廉洁政府

政府要为民。坚持以人为本、执政为民，体察社情民意，倾听群众呼声，关心群众疾苦，切实解决人民群众最关心、最直接、最现实的利益问题，使发展振兴成果更多惠及人民群众。深入推进政务公开，切实保障人民群众的知情权、参与权、表达权和监督权。全面推进依法行政，建设法治政府，切实提高政府的执行力和公信力。自觉接受市人大及其常委会的法律监督和工作监督，自觉接受市政协的民主监督和社会的公众监督，认真办理人大代表议案、建议和政协提案，不断提高办结率和满意率。随时受理代表和委员的建议和意见，广泛听取各民主党派、工商联、无党派人士和各人民团体、社会各界的建议和意见。

政府要务实。坚持深入实际，调查研究，做到问政于民、问需于民、问计于民。树立过紧日子思想，艰苦奋斗，勤俭节约，把有限的资金用在促进发展上，用在改善民生上。坚持说实话、办实事、求实效，提倡开短会、发短文、讲短话，多做打基础、利长远的工作，着力解决影响改革发展稳定的突出问题。认真负责，敢于碰硬，遇到困难不退缩，碰到矛盾不回避。切实把心思放在发展上，把气力用在落实上，把项目一个一个抓实，把事情一件一件办好，努力做出经得起实践、人民、历史检验的实绩。

政府要高效。深化行政审批制度改革，提高行政效能，坚决杜绝一件事情让群众跑来跑去、领导批来批去、部门转来转去的推诿扯皮现象。进一步优化发展环境，每个政府工作人员都要做到决不让工作在我这里被延误，决不让客商在我这里遭冷落，决不让政府形象在我这里受影响。完善政府绩效考评体系，严格执行首问负责制、限时办结制和责任追究制，提高办事效率，提供一流服务，创造一流业绩。

政府要廉洁。坚持从严治政，强化权力监管，加强公共资源交易系统建设，加大对工程建设、土地出让、政府采购、工程招标、国有资产处置和大额资金使用等重点领域的行政监察和审计监督力度，确保权力在阳光下运行。坚决纠正损害人民群众利益的不正之风，严肃查处各类违法违纪案件，决不让腐败分子有藏身之地，决不让腐败问题损害鞍山的整体形象，决不让腐败问题影响鞍山的振兴大业。

各位代表，我们面临的任务艰巨而繁重，我们肩负的责任重大而光荣。让我们在市委的领导下，在市人大、市政协监督支持下，团结带领全市各族人民，锐意进取，扎实工作，以更顽强的斗志、更务实的作风、更有力的措施，为实现鞍山老工业基地科学发展、创新发展、和谐发展而努力奋斗！
